# Global Change Sharpens the Double-Edged Sword Effect of Aquatic Alien Plants in China and Beyond

**DOI:** 10.3389/fpls.2019.00787

**Published:** 2019-06-12

**Authors:** Hao Wu, Jianqing Ding

**Affiliations:** ^1^College of Life Sciences, Xinyang Normal University, Xinyang, China; ^2^School of Life Sciences, Henan University, Kaifeng, China

**Keywords:** alien aquatic plants, biological invasions, aquatic ecosystem, global change, China

## Abstract

Many alien aquatic plants are deliberately introduced because they have economic, ornamental, or environmental values; however, they may also negatively affect aquatic ecosystems, by blocking rivers, restricting aquatic animals and plants by decreasing dissolved oxygen, and reducing native biodiversity. These positive and/or negative ecological effects may be enhanced under global change. Here, we examine the impacts of global change on aquatic alien plant introduction and/or invasions by reviewing their introduction pathways, distributions, and ecological effects. We focus on how climate change, aquatic environmental pollution, and China’s rapid economic growth in recent decades affect their uses and invasiveness in China. Among 55 species of alien aquatic plants in China, 10 species are invasive, such as *Eichhornia crassipes*, *Alternanthera philoxeroides*, and *Pistia stratiotes*. Most of these invaders were intentionally introduced and dispersed across the country but are now widely distributed and invasive. Under climate warming, many species have expanded their distributions to areas where it was originally too cold for their survival. Thus, these species are (and will be) considered to be beneficial plants in aquaculture and for the restoration of aquatic ecosystems (for water purification) across larger areas. However, for potential invasive species, climate warming is (and will be) increasing their invasion risk in more areas. In addition, nitrogen deposition and phosphorus inputs may also alter the status of some alien species. Furthermore, climate warming has shifted the interactions between alien aquatic plants and herbivores, thus impacting their future spreads. Under climate change, more precipitation in North China and more frequent flooding in South China will increase the uncertainties of ecological effects of alien aquatic plants in these regions. We also predict that, under the continuing booming economy in China, more and more alien aquatic plants will be used for aquatic landscaping and water purification. In conclusion, our study indicates that both human activities under rapid economic growth and climate change can either increase the potential uses of alien aquatic plants or make the aquatic invaders worse in China and other areas in the world. These findings are critical for future risk assessment of aquatic plant introduction and aquatic ecosystem restoration.

## Introduction

Global change includes climate change, nitrogen deposition, changes in land-use patterns, and biological invasions (Lin et al., 2010; Pyšek et al., 2010; Lu et al., 2013). Global change may accelerate the spread of alien plants, alter the species composition of plant communities, and affect the physiological and/or ecological traits of alien plants in aquatic ecosystems (Maki and Galatowitsch, 2004; Hastwell et al., 2008; Sorte et al., 2013; Henriksen et al., 2018). For instance, with rapidly growing international trades, many aquatic alien plants from around the world have been intentionally or unintentionally introduced into China (Ding et al., 2008; Wang et al., 2016), and some of them have become invaders for many reasons, such as the lack of co-evolved natural enemies (Lu et al., 2013; You et al., 2014). More seriously, biological invasions, as a major component of global change, have caused significant ecological and economic impacts on aquatic ecosystems, together with the impacts of other factors, such as global warming, eutrophication, and flooding (Hastwell et al., 2008; Collinge et al., 2011; Thouvenot et al., 2013; Lu et al., 2015).

Aquatic plants, as an ecological group closely dependent on water, have multiple ecotypes, including emergent, floating-leaved, submersed, and free-floating forms, totaling more than 2600 species of aquatic plants belonging to 88 families in the world (Chambers et al., 2008; Li, 2014). Although aquatic plants only account for approximately 2% of the 350,000 angiosperm species, they play key roles in the functioning of aquatic ecosystems (Carpenter and Lodge, 1986; Jeppesen et al., 1998; Hilt et al., 2017). Due to the growing ornamental, horticultural, and aquacultural trades and/or unintentional transport, many aquatic plants have been introduced into new continents or countries from their native range and have become alien species (Ding et al., 2008; Hussner, 2012). Unlike the immobile roots of their terrestrial congeners, aquatic alien plants usually have relatively weaker root systems, and some of them are free-floating macrophytes; thus, the fluctuating water further provides suitable conditions for the dispersal and diffusion of these alien diaspores, especially during flooding (Li, 2014).

Aquatic alien plants may have significant double-edged sword effects with respect to ecology. On the one hand, they provide numerous ecosystem services, including ornamental, landscaping, ecological restoration, food, forage, and green manure uses (Hussner, 2012; Wang et al., 2016). For example, many aquatic alien species of Nymphaeaceae and Alismataceae are introduced into China from America and Europe for use as ornamental or aquarium plants, such as *Victoria regia* and *Echinodorus amazonicus* (Chen et al., 2012). On the other hand, aquatic invasive plants have been shown to cause more serious impacts on their habitats than their terrestrial counterparts (Vila et al., 2009). In freshwater ecosystems especially, once aquatic alien plants successfully become invaders, they hinder river runoff, cause oxygen deficiency, reduce water quality and native biodiversity, and even disturb food web structures (Hussner, 2012; Hussner et al., 2017; Kennedy and El-Sabaawi, 2017; Liu D.S. et al., 2017). Relative to their native accompanying species, many aquatic invaders are opportunistic species that could quickly capitalize on increased resources, thus their growth and reproduction may be enhanced by elevated temperature and precipitation (Blumenthal, 2006; Sorte et al., 2013), which could ultimately alter their positive or negative ecological effects under rapid global change in the future. Therefore, examining the ecological effects of aquatic alien plants under global change is crucial for utilizing biological resources, predicting and preventing aquatic invasions, and protecting native biodiversity in freshwater ecosystems; however, such work has rarely been reported.

Here, we focus on how climate change, aquatic environmental pollution, and China’s rapid economic growth in recent decades affect alien aquatic plant uses and invasiveness. China has been experiencing a booming economy and has greatly increased international trades in the past 40 years, which dramatically facilitates aquatic alien plant establishment across this country (Ding et al., 2008; Weber and Li, 2008; Wu et al., 2010). Additionally, there are many types of freshwater bodies (lakes, rivers, estuaries, ponds, etc.) in China, further benefiting the spread and diffusion of aquatic alien plants (Wang et al., 2016). For example, the water hyacinth, *Eichhornia crassipes*, a free-floating aquatic macrophyte native to South America, was initially introduced into China for its ornament value (Qin et al., 2016a), and it also has water purifying properties in many large freshwater bodies of China (Wang et al., 2012, 2013; Liu et al., 2015). However, due to the booming economy and industrial development of China, high amounts of nutrients have been largely deposited into freshwater, accelerating eutrophication (Ding et al., 2008) and facilitating *E. crassipes* growth, which has made this plant the most important aquatic invasive plant in South China (Ding et al., 2006; You et al., 2014). Furthermore, the increasing global ornamental trades and developed hydrographic networks of China have also accelerated *E. crassipes* invasion and dispersal (Gao and Li, 2004; Liu D.S. et al., 2017). As one of the world’s worst aquatic weeds, *E. crassipes* has widely invaded into the rest of the world besides China, e.g., in Southeast Asia, Southeastern United States, Central America, and Central and Western Africa, causing serious damages to environment, biodiversity, economy, and human health in invaded regions (Ding et al., 2006; Villamagna and Murphy, 2010).

China is a geographically vast country, spanning 50 degrees of latitude and covering five climatic zones (Wang et al., 2016); thus, with climate change, high latitudinal regions of North China have experienced a larger temperature rise in the last 60 years, and this warming process will continue (Yang et al., 2018). In addition, the precipitation of high latitudes in North China will also increase in the future (Gao et al., 2015). These climate changes may accelerate the distribution of aquatic alien plants into higher latitudes in China. For example, the alligator weed *Alternanthera philoxeroides*, one of China’s major aquatic invaders native to South America that was introduced as a fodder species in the 1930s, has now expanded 2° of its northern boundary (from 34.7°N to 36.8°N) along latitudinal gradients in the last decade (Lu et al., 2015). Warming could expand the range of both *A. philoxeroides* and its natural enemy *Agasicles hygrophila*, an insect introduced for its biological control, shifting their interactions, and likely facilitating its invasiveness (Lu et al., 2013, 2015; Wu et al., 2017a). Together, under rapid global change, aquatic ecosystems would suffer a greater threat from biological invasions (Wu and Ding, 2014), and the double-edged sword of the ecological effects of aquatic alien plants may be further sharpened.

Here, we review the impacts of global change on aquatic alien plants and focus on the geographic origins and introduction pathway of these plants in China. We hypothesize that (1) global change has significant impacts on the double-edged sword effects of aquatic alien plants across the world and (2) specifically, the negative ecological effects of China’s aquatic alien plants will be aggravated by global change.

## Aquatic Alien Plants in China

In conjunction with previous related studies, we define aquatic alien plants as plant species that were introduced from their origins into new countries or regions due to intentional or inadvertent human involvement; the life cycles of these plant species are almost completely dependent on the water, or these plants are submerged for at least one part of their life history (Cook, 1990; Pyšek et al., 2004; Hussner, 2012; Li, 2014; Wang et al., 2016). By contrast, aquatic invasive plants are species among aquatic alien plants that “cause, or have the potential to cause, harm to the environment, economies, or human health” (GISP, 2003). We only study aquatic alien plants that occur in freshwater habitats and exclude species varieties by artificially breeding.

**FIGURE 1:**
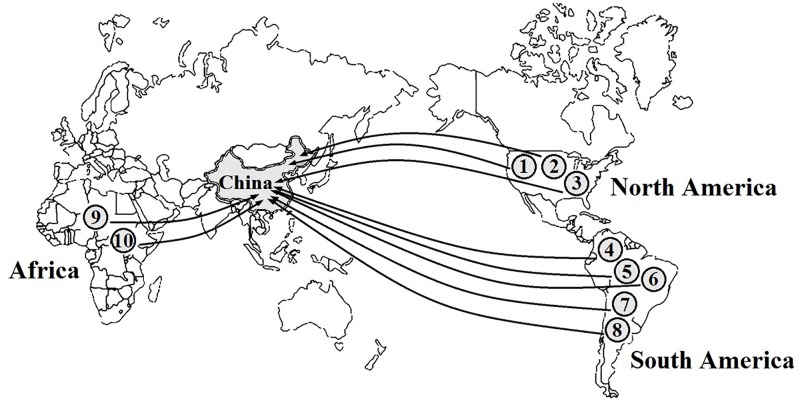
Geographical origins of 10 major aquatic invasive plants in China. Data are from the announcements of “*Inventory of Invasive Species in China* (first–fourth volumes)” which were enacted by the Ministry of Ecology and Environment of China & Chinese Academy of Sciences (2003, 2010, 2014, 2016) (http://www.mee.gov.cn/) and some published literatures (Ding et al., 2008; Yan et al., 2014; Wang et al., 2016). Latin names of species code in this figure are *Cabomba caroliniana* (1), *Spartina alterniflora* (2), *Hydrocotyle vulgaris* (3), *Azolla filiculoides* (4), *Alternanthera philoxeroides* (5), *Myriophyllum aquaticum* (6), *Eichhornia crassipes* (7), *Pistia stratiotes* (8), *Brachiaria brizantha* (9), and *Brachiaria mutica* (10).

**FIGURE 2:**
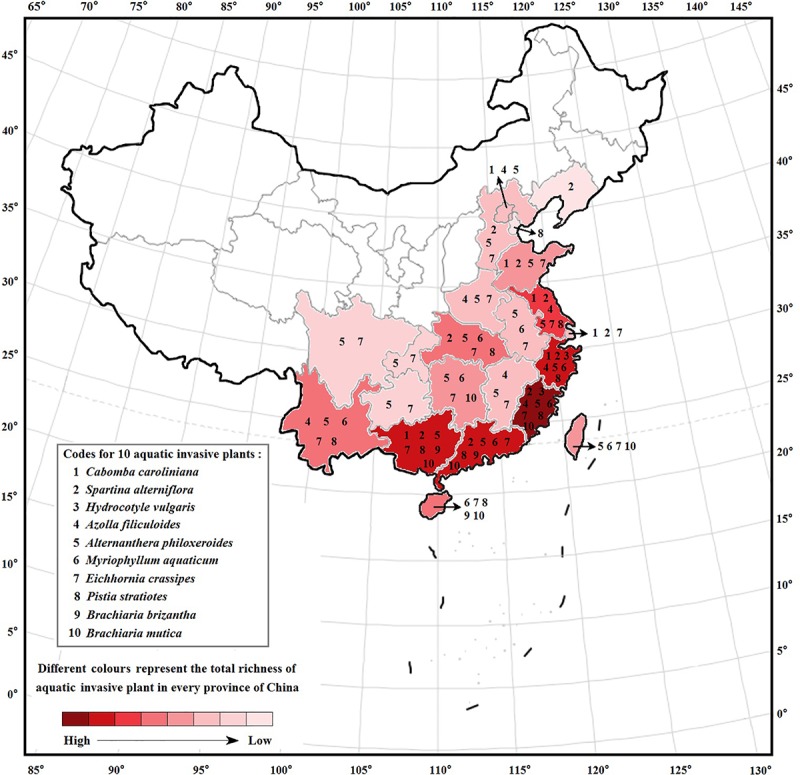
Interprovincial distributions of China’s 10 major aquatic invasive plants. Data are from the Chinese books including “*The Chinese Aquatic Plants*” (Chen et al., 2012), “*The Checklist of the Invasive Plants*” (Ma, 2013), “*Inventory Invasive Alien Species in China*” (Xu and Qiang, 2007), and “*Illustrations of Alien Invasive Plants in China*” (Yan et al., 2016); Chinese databases including “Chinese Virtual Herbarium” (http://www.cvh.ac.cn/), “Flora Reipublicae Popularis Sinicae” (the online version, http://frps.eflora.cn/), and “National Specimen Information Infrastructure of China” (http://mnh.scu.edu.cn/); and some published literatures (Ren et al., 2004; Ding et al., 2008; Zhang and Meng, 2013; Yan et al., 2014; Wang et al., 2016; Wu et al., 2016).

In total, 55 aquatic alien plant species belonging to 20 families and 29 genera were recorded in freshwater ecosystems in China (Supplementary Table 1). Nymphaeaceae had the highest species number of aquatic alien plants (11 spp.), followed by Alismataceae with 10 species and Gramineae with 7 species, while the other 11 families only possessed 1 alien plant species. According to our survey, 55 aquatic alien plant species were intentionally introduced into China through human involvement, and the original purposes were to use them for ornamental, aquatic landscaping, water purification, and forage purposes. However, 10 species among them (nearly 18%) later became invaders, i.e., *Cabomba caroliniana*, *Spartina alterniflora*, *Hydrocotyle vulgaris*, *Azolla filiculoides*, *A. philoxeroides*, *Myriophyllum aquaticum*, *E. crassipes*, *Pistia stratiotes*, *Brachiaria brizantha*, and *Brachiaria mutica*. Among these, five species were introduced from South America, three species were introduced from North America, and two species were introduced from Africa (Figure 1). One of the worst invaders, *A. philoxeroides*, has invaded 18 provinces spanning a large latitudinal gradient, followed by *E. crassipes*, which has invaded 16 provinces (Figure 2). *S. alterniflora*, *P. stratiotes*, and *M. aquaticum* also have wide distributions and occur in more than nine provinces. Compared with inland provinces, there are more diverse aquatic invasive plants in the coastal provinces of China, such as Fujian, Guangdong, Guangxi, Zhejiang, and Jiangsu provinces. Moreover, the richness of aquatic invasive plant species in China increases toward further east and south (Figure 2). This pattern is consistent with the latitudinal diversity gradient (LDG) rule which states that global biodiversity usually declines from tropics to the poles (Fischer, 1960), as the solar radiation and precipitation decrease with increasing latitudes (Lu et al., 2016; Wu et al., 2016). Thus, the low average annual temperature in higher latitude would also be unfavorable for the growth and reproduction of China’s aquatic invasive plants, most of which are native to the tropics with a long history of adaptation to higher temperatures.

## Positive and Negative Impacts of Global Change on Aquatic Alien Plants

Aquatic alien plants have caused significant double-edged sword effects on the freshwater ecosystems. They may threaten human health by providing habitats for mosquitoes (O’Meara et al., 2003; Chandra et al., 2006). They also hamper recreational activities and disrupt agricultural production, causing great economic losses (Oreska and Aldridge, 2010; Rumlerova et al., 2016; Keller et al., 2018; Tanveer et al., 2018). Alien aquatic plants often compete for space, nutrients, and sediment fertilities with native macrophytes, thus hindering their re-establishment and decreasing diversity (Michelan et al., 2018; Silveira et al., 2018). Some of them aggravate water loss in invaded habitats through extensive transpiration (Fraser et al., 2016); accelerating water pollution by increasing sedimentary organic matter (Cuassolo et al., 2016; Bertrin et al., 2017); and reducing oxygen diffusion across the water–air interface (Chamier et al., 2012), etc.

Alien aquatic plants could also increase regional or local aquatic plant species richness (Gołdyn, 2010; Bolpagni and Piotti, 2015). As ecosystem engineers, they could be used for water purification by reducing water turbidity, decreasing sediment nutrient loading, and intensifying seasonal fluctuations of oxygen and carbon for keeping the balance of benthic nutrients in freshwater ecosystems (Thomaz et al., 2015; Bai and Shang, 2017; Ribaudo et al., 2018). They may also provide shelters for aquatic macroinvertebrates (Rocha-Ramírez et al., 2014) and increase pollinator visitants for native aquatic plants (Stiers et al., 2014), etc.

**FIGURE 3:**
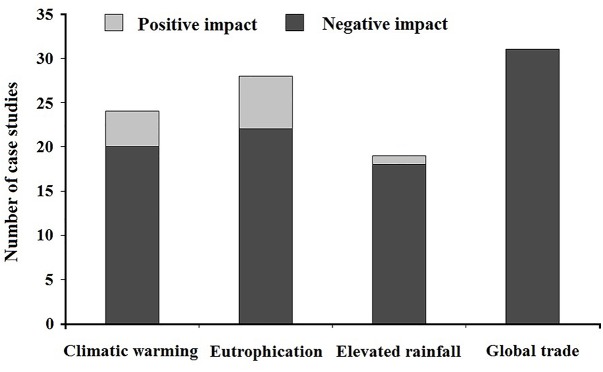
Impacts of global change on the ecological effects of aquatic alien plants at the global scale. Data are from the related 102 published studies that are listed in the Supplementary Material (see Supplementary Table 2).

In this study, to examine the impacts of rapid global change on both of the negative and positive effects of aquatic alien plants, we searched the literatures (article or review) from the ISI Web of Science database, mostly published during 1998–2018s, using the following search terms: (“warming” or “temperature” or “climate warming” or “global warming”), (“eutrophication” or “nitrogen” or “phosphorus”), (“flood” or “precipitation” or “rainfall”), (“trade” or “global trade” or “economic globalization”) combined with (“aquatic alien plant” or “alien aquatic plant” or “aquatic exotic plant” or “exotic aquatic plant”). We carefully selected the articles that clearly reported the positive or negative impacts of global change on ecological effects of aquatic alien plants and excluded studies that addressed unrelated topics. In total, we collected data from 102 case studies about the impacts of global change on aquatic alien plants at a global scale (see Supplementary Table 2, the reference list). We found that, most of studies on climatic warming, eutrophication and elevated rainfall reported negative impacts and a few addressed positive impacts, while studies for global trade only dealt with the negative impacts, i.e., ecological effects of aquatic alien plants (Figure 3).

**FIGURE 4:**
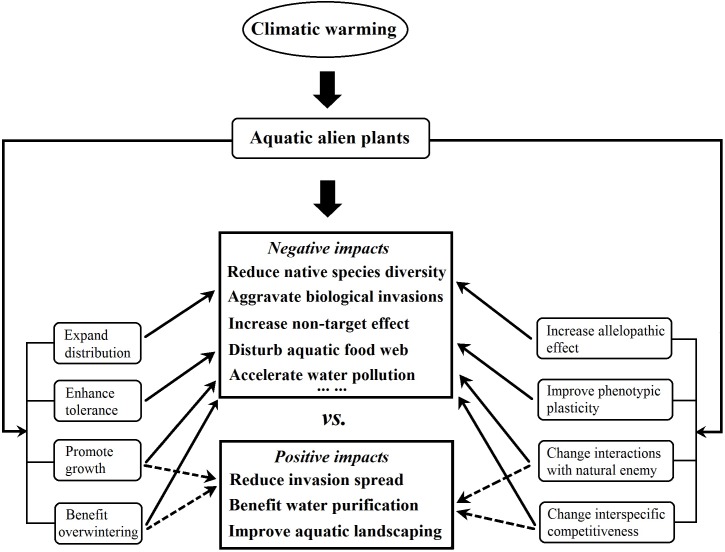
Positive and negative impacts of climatic warming on the ecological effects of aquatic alien plants. The thin solid arrow indicates that climatic warming will facilitate the negative ecological effects of aquatic alien plants, while the thin dotted arrow indicates that warming will facilitate their positive ecological effects. Data are from the related 24 published studies that are listed in the Supplementary Material (see Supplementary Table 2).

One of the positive impacts of climatic warming on aquatic alien plants is water purification through an increase in the inhibition by certain aquatic alien plants of harmful algae, and through improving aquatic landscaping, as a result of increasing in adaptability of alien ornamental plants to aquatic environments. The negative impacts of warming include the reduction in native species diversity through increased interspecific competition, accelerated water pollution through increased alien plant litter decay rates, and aggravated aquatic alien plant invasions through an increase in their biomass and overwintering (Figure 4). Some aquatic invaders such as *P. stratiotes* and *E. crassipes* are floating plants and overwinter with floating vegetative tissues; the warmer water temperature could prevent the leaves and roots from being killed by frost in the winter, and their overwintering vegetative biomass respond quickly to the elevated temperatures, thus climate change will enhance their invasion and increase their negative impacts (Santos et al., 2011; Hussner et al., 2014; You et al., 2014).

Eutrophication increases the applicability of aquatic alien plants for water purification and can decrease the relative competitive ability of certain aquatic invasive plants compared with their native accompanying plants (Qin et al., 2016b; Xu et al., 2017). However, eutrophication strongly benefits the reproduction, clonal spread, compensatory growth, metabolic enzyme activity, and nutrient assimilation of many aquatic invasive plants around the word, which would further aggravate their invasiveness (Coetzee et al., 2011; Li and Wang, 2011; You et al., 2014).

Elevated rainfall was reported to reduce the salinity and increase the water level of Lake Naivasha in Kenya, which was favorable for the survival and establishment of several exotics such as *Cyperus papyrus* and *Potamogeton distinctus*, improved the native biodiversity (Lamb et al., 2003). However, the flooding caused by elevated rainfall accelerates the downstream movement of seedlings or propagules of alien plants and may wash away many small native hydrophytes, resulting in a decrease in the resistance of freshwater ecosystems to invasion (Holmes et al., 2008; Hofstra et al., 2010; Wu et al., 2017b). Extreme hydrological events also increase the landscape connectivity of water bodies in aquatic ecosystems, increasing external nutrient inputs in freshwater discharged from agricultural areas and sewage effluents and thus accelerating aquatic invasions (Espinar et al., 2015; Anufriieva and Shadrin, 2017).

Global trade (particularly aquarium and ornamental trade) has been identified as the major pathway for aquatic alien plant introductions, and the rapid spread of propagules and/or seedlings of nonindigenous aquatic plants that caused by trade may accelerate their invasions worldwide (Ding et al., 2008; June-Wells et al., 2012; Oele et al., 2015). High numbers of potential aquatic invaders have been massively introduced into many countries for sale through global trade without strict legal regulations (Thum et al., 2012). Global trade also facilitates mutualistic invasion; for instance, the invasive *Physa acuta* was carried into Thailand and Laos in the introduction process of many alien aquatic ornamental/invasive plants (Ng et al., 2018). Furthermore, with the rapid development of global online trade, alien aquatic ornamental plants can be bought online easily and cheaply through the unregulated market, and some of them are invaders, such as the notorious *E. crassipes* (Brundu et al., 2013). In general, the negative impacts of global change on aquatic alien plants are much greater than its positive impacts at a global scale.

## Warming Is Expanding the Distributions of Aquatic Alien Plants Across China

The Earth’s climate has warmed by nearly 1.0°C over the past 100 years, and it is predicted that the average temperature will continue to increase by 3°C at the end of the 21st century, however, it could breach 1.5°C between 2030 and 2052 if climate warming continues at its current rate (IPCC, 2018; Tollefson, 2018). In China, the average temperature has increased by approximately 0.4°C per decade in the last 40 years, and the high latitudinal regions in North China will have a greater temperature increase under global warming (Walther et al., 2002; Ding et al., 2007). Increasing water temperature has caused profound impacts on the establishment, growth, phenology, and distribution of aquatic plant species in freshwater ecosystems, especially alien species, because aquatic alien plants usually have more active responses to climatic warming compared with native co-occurring plants (Sorte et al., 2013; Hussner, 2014; You et al., 2014). Thus, warming may increase the risk of aquatic alien plants transforming into invaders, such as the alien *Thalia dealbata* in China (Chen and Ding, 2011), and accelerate aquatic invasive plants spreading to more new regions (especially higher latitudes), such as the invasive *A. filiculoides* in Spain, *P. stratiotes* in Germany, and *Egeria densa* in the United States; the invasiveness and overwintering of these invaders were greatly promoted by warming (Santos et al., 2011; Hussner et al., 2014; Espinar et al., 2015).

**FIGURE 5:**
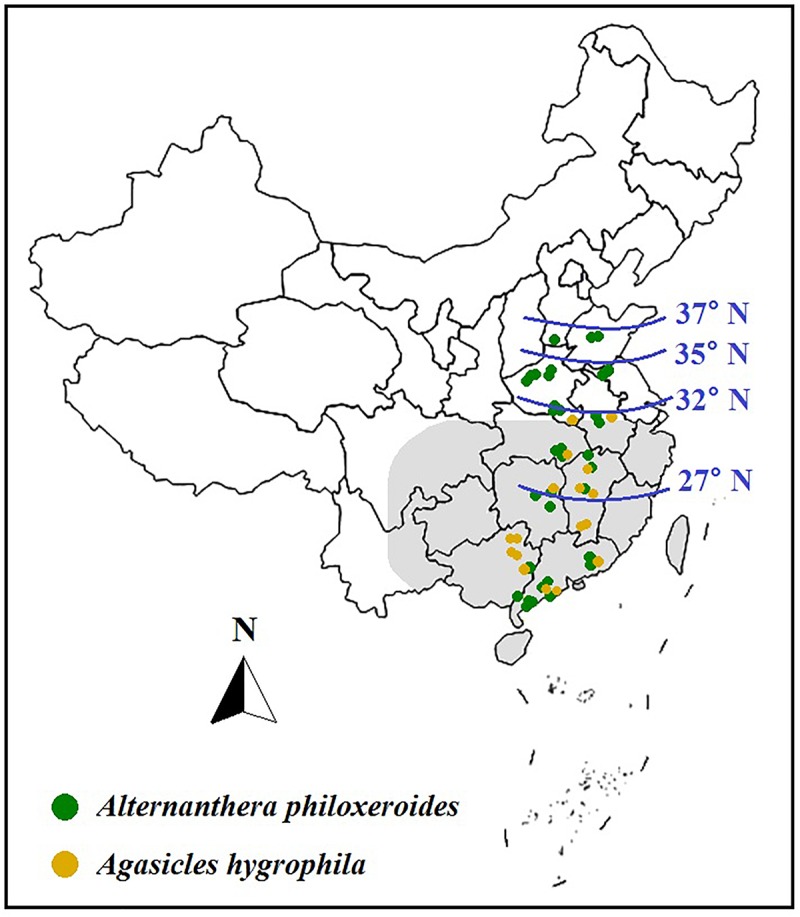
Dynamic distributions of aquatic invasive *A. philoxeroides* and its biocontrol beetle *Agasicles hygrophila* in China under global warming. In 1995, the potential northern boundaries of *A. philoxeroides* and *A. hygrophila* were predicted to be at 31.5°N (as the shadowing shows) and 27°N, respectively. In 2001, the northern boundaries of *A. philoxeroides* and *A. hygrophila* were found at 32°N and 35°N, respectively. In 2013, the northern boundary of *A. hygrophila* was still limited at 32°N (as the yellow dots show); however, *A. philoxeroides* had expanded to approximately 37°N (as the green dots show). Data were from related literatures (Julien et al., 1995; Ma, 2001; Lu et al., 2013).

In China, climate warming has also increased the net photosynthetic rate and morphological plasticity of invasive *A. philoxeroides* (Lu et al., 2013; Chu et al., 2014; Wang et al., 2017), as well as accelerated its spread to higher latitudes of North China. Warming also increased enemy release from the bio-control beetle *A. hygrophila* (Figure 5), because *A. philoxeroides* tolerated cold better than its natural enemy *A. hygrophila* and expanded more fast to the higher latitudes, while *A. hygrophila* failed to overwinter in the low temperature of Northern China, thus, geographical gap between *A. philoxeroides* and *A. hygrophila* would be shifted to higher latitudes under warming, further benefiting plant invasion (Lu et al., 2013). However, warming could also affect the biotic interactions among *A. hygrophila*, *A. philoxeroides*, and its native congener *A. sessilis* in China, as warming significantly increased the plant performances (e.g., aboveground biomass, flower, and seed numbers, etc.) of *A. sessilis* relative to the co-occurring invader in the presence of *A. hygrophila*, and the beetle abundance on *A. philoxeroides* was higher than that on *A. sessilis* under elevated temperature, thus relatively increased the biotic resistance of native *A. sessilis* to *A. philoxeroides* invasion (Lu et al., 2016).

Warming significantly increases the growth of *E. crassipes*, which is one of the major aquatic invaders in China (You et al., 2014). Consistent with findings in other countries (Hussner, 2014; Espinar et al., 2015; Vojtkó et al., 2017), invasion by *P. stratiotes*, *A. filiculoides*, and *C. caroliniana* in China would also be continuously aggravated by climatic warming. According to the latest survey, *C. caroliniana* has expanded its northern distribution boundary to the 40°N region of Beijing City, i.e., at higher latitudes (Zhang and Meng, 2013). Warming could also improve the adaption of some aquatic alien plants used in landscaping (such as *Nymphaea rubra*) and thus increase the species diversity of aquatic vegetation (Hussner and Lösch, 2005; Vojtkó et al., 2017). Warming could even weaken the spread of certain aquatic invasive plants, such as *Elodea canadensis* in Polish lakes, as warming strengthened the thermal stratification of water columns and thus might reduce the nutrient cycles between deeper waters and surface, which would weaken the nutrient supply to *E. canadensis* (Kolada and Kutyła, 2016; Vasconcelos et al., 2019). Furthermore, the asymmetry of global warming between day and night may further exacerbate the invasion of aquatic alien plants and cause considerable detrimental impacts (Harvey, 1995; Walther et al., 2002; Chu et al., 2014). For example, the submerged plant species *Myriophyllum spicatum*, which is native to Europe, Asia, and has invaded to North America, has prolonged its growing season under climate warming, thus the increasing abundance in freshwater ecosystems significantly increased its biomass and carbon stock (Velthuis et al., 2018). In China, many aquatic invaders were introduced from the tropics with higher thermal tolerance, and they are distributed in large geographical ranges of China (Figure 1, 2), warming may increase their invasion risk at high latitudes in the future.

## Eutrophication Promotes the Applicability and Invasion Risks of Alien Hydrophytes

Water eutrophication is the consequence of human activities caused by depositing high levels of organic compounds and/or nutrients (nitrogen and phosphorus) into freshwater ecosystems (Carpenter et al., 1998; Yu et al., 2018). In China, a large amount of domestic and industrial wastewater is discharged without treatment, which seriously intensifies eutrophication (Ding et al., 2008; Le et al., 2010). At present, more than 70% of the major lakes in China have undergone severe eutrophication (Jin et al., 2005; Yu et al., 2018). For these reasons, there is increased demand for certain aquatic alien plant for water purification in China, such as *E. crassipes*, *P. stratiotes*, *M. aquaticum*, *A. philoxeroides*, *T. dealbata*, *S. alterniflora*, which all have excellent phytoremediation properties, with a high nutrient removal ability and allelopathic inhibition of harmful algae (Zuo et al., 2012; Qin et al., 2016a); consequently, water transparency and the control of cyanobacterial blooms could increase.

On the other hand, the growth rates, photosynthetic capacity, and phenotypic plasticity of aquatic alien plants usually increase more intensively than those of native hydrophytes in nutrient-rich waters (Riis et al., 2010; Yu et al., 2018), and most aquatic alien plant species become successful invaders. In China, eutrophication increases biomass accumulation, asexual reproduction, compensatory growth, and photosynthetic leaf areas of *A. philoxeroides* (Shen et al., 2007; Jiang et al., 2010; Zuo et al., 2012; Ding et al., 2014); promotes the growth rate, clonal propagation, nitrate reductase, and glutamine synthetase activities of *E. crassipes* (Li et al., 2008, Li and Wang, 2011); and increases the biomass, shoot length, and nutrient uptake of *M. aquaticum* (You et al., 2013; Liu S.B. et al., 2017), which intensify the interspecific competition of these three aquatic invaders with respect to native plants. In addition, interactions between warming and eutrophication have been shown to significantly improve the overwintering ability of *E. crassipes* in China (You et al., 2014). Excessive growth of aquatic invasive plants in nutrient-rich waters may exacerbate the deposition of their dead matter and reduce the dissolved oxygen in invaded habitats. Indeed, more than a hundred rivers with eutrophication have been blocked by *E. crassipes* and/or *A. philoxeroides* in southern China over the last 30 years (Ding et al., 2008). Similarly, eutrophication in other countries also severely accelerates aquatic plant invasions, such as *E. canadensis* in New Zealand and *Glyceria maxima* in Australia (Loo et al., 2009; Riis et al., 2010). Moreover, the annual bulk deposition of nitrogen in China has increased by 8 kg per hectare in the last 30 years (Liu et al., 2013); excessive nitrogen flowing into waters would aggravate the eutrophication of freshwater ecosystems and thus further accelerate China’s aquatic plant invasions.

## Increasing Rainfall Accelerates the Spread of Aquatic Alien Plants in China

With ongoing climate change, extreme global climatic events become more frequent, such as floods, and China is under a high risk of heavy rainfall (Piao et al., 2010; Diez et al., 2012). For instance, summer precipitation has significantly increased in southern China since the 1960s, which readily causes severe flooding (especially in the Yangtze River basin) (Piao et al., 2010), while the frequency of moderate rain has increased in the high latitudes of northern China, and extreme rainfall may increase over most of China in the future (Gao et al., 2015). This elevated rainfall improves the adaptation and survival of aquatic alien ornamental/landscape plants in arid regions of northern China; however, this would thus promote the transport of alien plant propagules across China, particularly those of free-floating species (such as *E. crassipes*, *P. stratiotes*, and *A. filiculoides*), as well as provide more suitable aquatic environments for the spread and establishment of aquatic invasive plants at high latitudes. It has been reported that some aquatic plant invasions (e.g., *A. philoxeroides* and *M. aquaticum*) in China are significantly correlated with precipitation, as rainfall increases their biomass or species coverage (You et al., 2013; Wu et al., 2017b). For instance, elevated rainfall increased peroxidase and superoxide dismutase activities, species coverage, and new leaf numbers of *A. philoxeroides*, while water level fluctuation increases *A. philoxeroides* shoot length but reduces intraspecific competition (Yu, 2011; Chen et al., 2016). Elevated rainfall/water level fluctuations can also increase clonal integration, the number of branches, and the stolon length of the invasive species *M. aquaticum* (You et al., 2013; Chen et al., 2016), facilitating its invasion in China. Flash flooding due to elevated rainfall also reduces the biotic resistance of native aquatic plants, aggravates eutrophication through fertilizer runoff, and improves the connectivity of waters, increasing the invasion of freshwater ecosystems by aquatic alien plants (Collinge et al., 2011; Espinar et al., 2015; Anufriieva and Shadrin, 2017). In Florida, elevated rainfall increases the water content in the leaf axils of aquatic alien plants, which improves habitats for the growth of mosquitoes and thus exacerbates potential harms caused to human health (O’Meara et al., 2003). In addition, large-scale hydraulic projects, such as China’s South-to-North Water Diversion Project (SNWD), may provide an express channel for the spread of aquatic invasive plants, especially during flooding (Liu D.S. et al., 2017), and elevated rainfall could even interact with climatic warming to accelerate the spreading of aquatic invaders (Espinar et al., 2015). Together, these findings indicate that rainfall will increase the uncertainties of the ecological effects of alien aquatic plants.

## Booming Global Trade Aggravates China’s Ongoing Aquatic Invasions

Since China implemented the landmark “reform and opening” policy in 1978, its gross volume of import and export trade increased rapidly. After China joined the World Trade Organization (WTO) in 2001, it has become the second largest importing country in the world, such that the total value of China’s imports and exports increased from approximately RMB 35.5 billion to RMB 27,800 billion over the period 1978–2017, which is a 782-fold increase, and its trading partners expanded from 40 to 231 countries/regions (National Bureau of Statistics of China [NBSC], 2017). Under global change, such phenomenal growth in the international trade of China also aggravated aquatic plant invasions (Ding et al., 2008; Weber and Li, 2008). Global trade effectively promotes the distribution and spread of many of these species, through horticultural, ornamental, and aquarium trades; the dumping of ballast water and the burgeoning unregulated Internet trades; and many aquatic alien ornamental plants that come from international trades usually have higher growth rates, cold tolerance, and dispersal ability (Pemberton and Liu, 2009; Martin and Coetzee, 2011; Azan et al., 2015). For example, the invasive species *E. crassipes* and *C. caroliniana* were introduced into China through global ornamental and aquarium trades without risk assessment and have subsequently severely invaded and threatened native plant diversity in Southern China (He et al., 2011; You et al., 2014). Other aquatic alien plants, such as *T. dealbata* and *E. densa*, which also have great potential invasiveness, are still imported into China through international trading (Chen and Ding, 2011). Recently, China started another huge international trade project, “The Belt and Road (B&R),” in 2013. Under this project, the total value of China’s imports and exports from the B&R reaches RMB 3.32 billion in 5 years, with a growth rate that is 1.4% higher than that of the national average level (National Bureau of Statistics of China [NBSC], 2017). These growing global trades may thus continuously increase the invasion risk of aquatic alien plants in China.

## Conclusion

Our study shows that alien aquatic plants have caused both positive and negative ecological effects on freshwater ecosystems and global change, such as climate warming, eutrophication, elevated rainfall, and global trade could increase or decrease those effects. In China, eutrophication increases the demands for aquatic alien plants for use in water purification and landscaping, while climate warming may improve the adaption of aquatic alien ornamental plants and even increase the biotic resistance of native plants to aquatic invaders. However, warming, eutrophication, and elevated rainfall could increase the invasiveness of many aquatic invasive plants, and booming global trade may accelerate the dispersal of aquatic invaders across this country. In brief, global change is sharpening the double-edged sword effect of China’s aquatic alien plants, increasing the utilization of aquatic alien plant resources, and aggravating their invasion risk.

Our study also indicates that human activities under rapid economic development and climate change can either accelerate aquatic alien plants establishment or the spread of invaders. It is necessary to intensify the risk assessment before introduction and prediction of the potential distributions of invading species. Our findings may assist in predicting aquatic plant invasions and the rational utilization of aquatic plant resources, as well as provide important implications for native plant biodiversity protection under rapid global change.

## Author Contributions

JD conceptualized the overall structure. HW collected and analyzed all the data. HW and JD wrote the manuscript and approved this final version of the manuscript to be published.

## Conflict of Interest Statement

The authors declare that the research was conducted in the absence of any commercial or financial relationships that could be construed as a potential conflict of interest.
