# Supplementary material for: Global Change Sharpens the Double-Edged Sword Effect of Aquatic Alien Plants in China and Beyond
Source: Front Plant Sci. 2019 Jun 12;10:787. doi: 10.3389/fpls.2019.00787 (PMC6582753; doi:10.3389/fpls.2019.00787)
Supplement: Supplementary file 1 [file Table_1.DOCX]

**Supplementary Table 1** The geographic origins, introduction pathway and purpose of aquatic alien plants in China. Data for this table are from the Chinese book ‘*The Chinese Aquatic Plants*’ (Chen et al., 2012), ‘*The Checklist of the Invasive Plants*’ (Ma et al., 2013), ‘*Inventory Invasive Alien Species in China*’ (Xu and Qiang, 2007), ‘*Illustrations of Alien Invasive Plants in China*’ (Yan et al., 2016), the ‘database of invasive alien species in China*’* (<http://www.chinaias.cn/>) and some related literature (Yan et al., 2012; Wang et al., 2016).

| **Family** | **Latin name** | **Geographic origins** | **Introduction pathway / Purpose** |
| --- | --- | --- | --- |
| Nymphaeaceae | *Victoria cruziana* | South America | intentional / ornamental, water purification |
|  | *Victoria regia* | South America | intentional / ornamental |
|  | *Victoria amazonica* | [Brazil](javascript:;) | intentional / ornamental, landscaping |
|  | *Nymphaea mexicana* | United States, Mexico, Australia | intentional / ornamental |
|  | *Nymphaea* *pygmaea* | Temperate region | intentional / ornamental, landscaping |
|  | *Nymphaea odorata* | United States | intentional / ornamental |
|  | *Nymphaea alba* | Europe | intentional / ornamental |
|  | *Nymphaea lotus* | [Egypt](javascript:;) | intentional / ornamental |
|  | *Nymphaea nouchali* | India | intentional / ornamental |
|  | *Nymphaea caerulea* | Egypt, Africa | intentional / ornamental |
|  | *Nymphaea gigantea* | [Australian](javascript:;) | intentional / ornamental |
| Alismataceae | *Echinodorus amazonicus* | [Brazil](javascript:;) | [intentional / aquarium](javascript:;) |
|  | *Echinodorus berteroi* | North America, Brazil | intentional / ornamental |
|  | *Echinodorus bleheri* | South America | [intentional / aquarium, ornamental, landscaping](javascript:;) |
|  | *Echinodorus cordifolius* | North America | intentional / ornamental |
|  | *Echinodorus grandiflorus* | Central America, Brazil | intentional / ornamental, aquarium, landscaping |
|  | *Echinodorus horizontalis* | South America | [intentional / aquarium](javascript:;) |
|  | *Echinodorus latifolius* | Central America, South America | [intentional / aquarium](javascript:;) |
|  | *Echinodorus major* | [Brazil](javascript:;) | intentional / ornamental, landscaping |
|  | *Echinodorus osiris* | [Brazil](javascript:;) | intentional / landscaping |
|  | *Echinodorus tenellus* | North America, South America | [intentional / aquarium](javascript:;) |
| Gramineae | *Oryza glaberrima* | Africa | intentional / cross breeding |
|  | *Spartina alterniflora* **(invasive)** | North America | intentional / ecological restoration, water purification |
|  | *Zizania palustris* | North America | intentional / ornamental, food, forage |
|  | *Zizania aquatica* | North America | intentional / ornamental |
|  | *Vetiveria zizanioides* | Mediterranean region | intentional / spiceberry, ecological restoration |
|  | *Brachiaria brizantha***(invasive)** | Tropical Africa | intentional / forage, green manure |
|  | *Brachiaria mutica* **(invasive)** | Tropical Africa | intentional / forage |
| Hydrocharitaceae | *Vallisneria americana* | North America | [intentional / aquarium](javascript:;) |
|  | *Vallisneria spiralis* | Tropical region | intentional / aquarium , ornamental |
|  | *Vallisneria gigantea* | New Guinea | intentional / aquarium , ornamental |
|  | *Vallisneria neotropicalis* | United States | intentional / aquarium , ornamental, landscaping |
|  | *Egeria densa* | South America | intentional / aquarium , ornamental |
| Aponogetonaceae | *Aponogeton madagascariensis* | [Madagascar](javascript:;) | intentional / ornamental, aquarium |
|  | *Aponogeton rigidifolius* | Sri Lanka | intentional / aquarium , ornament |
|  | *Aponogeton distachyos* | South Africa | intentional / ornamental, aquarium |
| Butomaceae | *Hydrocleis nymphoides* | South America | intentional / aquarium ,landscaping |
|  | *Limnocharis flava* | America | intentional / ornamental, forage |
| Cyperaceae | *Scirpus lacustris* | North America | intentional / ornamental, landscaping |
|  | *Cyperus alternifolius* | Africa | intentional / ornamental, landscaping |
| Pontederiaceae | *Eichhornia crassipes* **(invasive)** | South America | intentional / ornamental, water purification, forage |
|  | *Pontederia cordata* | South America | intentional / ornamental, landscaping |
| Marantaceae | *Thalia dealbata* | United States, Mexico | intentional / ornamental, water purification, landscaping |
|  | *Thalia geniculata* | America, Central Africa | intentional / ornamental, landscaping |
| Azollaceae | *Azolla filiculoides* **(invasive)** | South America | intentional / forage, green manure |
| Amaranthaceae | *Alternanthera philoxeroides* **(invasive)** | [Brazil](javascript:;) | intentional / forage, water purification |
| Araceae | *Pistia stratiotes* **(invasive)** | America | intentional / forage, water purification, landscaping |
| Cabombaceae | *Cabomba caroliniana* **(invasive)** | United States | intentional / aquarium, ornamental |
| Campanulaceae | *Lobelia cardinalis* | North America | intentional / ornamental, landscaping |
| Cruciferae | *Nasturtium officinale* | Europe | intentional / ornamental, landscaping |
| Haloragidaceae | *Myriophyllum aquaticum* **(invasive)** | Europe | intentional / landscaping, ornamental |
| Iridaceae | *Iris pseudacorus* | Europe | intentional / ornamental, landscaping |
| Nelumbonaceae | *Nelumbo lutea* | United States, Canada | intentional / ornamental |
| Typhaceae | *Typha latifolia* | America | intentional / ornamental, landscaping |
| Umbelliferae | *Hydrocotyle vulgaris* **(invasive)** | Europe, North America | intentional / ornamental, landscaping |

**REFERENCES**

Chen, Y. D., Ma, X. T., Du, Y. F., Feng, M., and Li, M. (2012) *The Chinese Aquatic Plants*. Zhengzhou: Henan Science and Technology Press.

Ma, J. S. (2013). *The Checklist of the Invasive Plants*. Bejing: Higher Education Press.

Wang, H., Wang, Q., Bowler, P. A., and Xiong, W. (2016). Invasive aquatic plants in China. *Aquat. Invasions.* 11, 1–9. doi: 10.3391/ai.2016.11.1.01

Xu, H. G., and Qiang, S. (2007). *Inventory Invasive Alien Species in China*. Beijing: Environmental Science Press.

Yan, J., Yan, X. L., and Ma, J. S. (2016) *Illustrations of Alien Invasive Plants in China.* Shanghai: Shanghai Science and Technology Press.

Yan, X. L., Shou, H. Y., and Ma, J. S. (2012). The problem and status of the alien invasive plants in China. *Plant. Divers. Resour.* 34, 287–313. doi: 10.3724/SP.J.1143.2012.12025
