# Supplementary material for: Global Change Sharpens the Double-Edged Sword Effect of Aquatic Alien Plants in China and Beyond
Source: Front Plant Sci. 2019 Jun 12;10:787. doi: 10.3389/fpls.2019.00787 (PMC6582753; doi:10.3389/fpls.2019.00787)
Supplement: Supplementary file 2 [file Table_2.DOCX]

**Supplementary Table 2.** Literature survey of [multiple](javascript:;) impacts of global change on aquatic alien plant at global scales. We searched the [relevant](javascript:;) [article](javascript:;)s with using a combination of search terms for aquatic alien plant and global change in the ISI Web of Science database (www.web of knowledge. com) at August 5, 2018. The specific search terms were as follows: TS=(((warming) or (temperature) or (climate warming) or (global warming) or (climatic warming)) and ((aquatic alien plant) or (alien aquatic plant) or (aquatic exotic plant) or (exotic aquatic plant))); TS=(((eutrophication) or (nitrogen) or (phosphorus)) and ((aquatic alien plant) or (alien aquatic plant) or (aquatic exotic plant) or (exotic aquatic plant))); TS=(((flood) or (precipitation) or (rainfall)) and ((aquatic alien plant) or (alien aquatic plant) or (aquatic exotic plant) or (exotic aquatic plant))); TS=(((trade) or (global trade) or (economic globalization)) and ((aquatic alien plant) or (alien aquatic plant) or (aquatic exotic plant) or (exotic aquatic plant))).

**Negative impacts of climatic warming (20 articles):**

| Adebayo, A. A., Briski, E., Kalaci, O., Hernandez, M., Ghabooli, S., Beric, S. B., Chan, F. T., Zhan, A., Fifield, E., Leadley, T., and MacIsaac, H. J. (2011). Water hyacinth (*Eichhornia crassipes*) and water lettuce (*Pistia stratiotes*) in the Great Lakes: playing with fire? *Aquat. Invasions*. 6, 1: 91–96. doi: 10.3391/ai.2011.6.1.11 |
| --- |
| Boggero, A., Basset, A., Austoni, M., Boggero, A., Basset, A., Austoni, M., Barbone, E., Bartolozzi, L., Bertani, I., Campanaro, A., Cattaneo, A., Cianferoni, F., Corriero, G., Dörr, A. M., …Fontaneto D. (2014). Weak effects of habitat type on susceptibility to invasive freshwater species: an Italian case study. *Aquat*. *Conserv*. 24, 841–852. doi: 10.1002/aqc.2450 |
| Espinar, J. L., Díaz-Delgado, R., Bravo-Utrera, M. A., and Vilà, M. (2015). Linking *Azolla filiculoides* invasion to increased winter temperature in the Doñana marshland (SW Spain). *Aquat. Invasions.* 10, 17–24. doi: 10.3391/ai.2015.10.1.02 |
| Hussner, A. (2014). Long-term macrophyte mapping documents a continuously shift from native to non-native aquatic plant dominance in the thermally abnormal River Erft (North Rhine-Westphalia, Germany). *Limnologica*. 48, 39–45. doi: 10.1016/j.limno.2014.05.003 |
| Hussner, A., and LoschR. (2007). Growth and photosynthesis of *Hydrocotyle ranunculoides* L. fil. in Central Europe. *Flora*, 202, 653–660. doi: 10.1016/j.flora.2007.05.006 |
| Hussner, A., Heidbuechel, P., Heiligtag, S. (2014). Vegetative overwintering and viable seed production explain the establishment of invasive *Pistia stratiotes* in the thermally abnormal Erft River (North Rhine-Westphalia, Germany). 119, 28–32. doi: 10.1016/j.aquabot.2014.06.011 |
| Hussner, A., Hofstra, D., Jahns, P., and Clayton, J. (2015). Response capacity to CO_2_depletion rather than temperature and light effects explain the growth success of three alien Hydrocharitaceae compared with native *Myriophyllum triphyllum* in New Zealand. *Aquat. Bot.*120, 205–211. doi: 10.1016/j.aquabot.2014.07.003 |
| Hyldgaard, B., and Brix, H. (2012). Intraspecies differences in phenotypic plasticity: Invasive versus non-invasive populations of *Ceratophyllum demersum*. *Aquat. Bot.* 97, 49–56. doi: 10.1016/j.aquabot.2011.11.004 |
| Kennedy, K. T. M., and El-Sabaawi, R. W. (2017). A global meta-analysis of exotic versus native leaf decay in stream ecosystems. *Freshwater. Biol*. 62, 1–13. doi:10.1111/fwb.12918 |
| Lu, X. M., Siemann, E., He, M. Y., Wei, H., Shao, X., and Ding, J. Q. (2015). Climate warming increases biological control agent impact on a non-target species. *Ecol. Lett.* 18, 48–56. doi: 10.1111/ele.12391 |
| Lu, X.M., Siemann, E., Shao, X., Wei, H., and Ding, J. Q. (2013). Climate warming affects biological invasions by shifting interactions of plants and herbivores. *Global. Change. Biol*. 19, 2339–2347. doi: 10.1111/gcb.12244 |
| [Myśliwy, M., and Szlauer-Lukaszewska A. (2017). Fern Azolla filiculoides at new sites in Oder river (Poland) -invader or ephemeral? Pol. J. Ecol. 65, 405–414. doi: 10.3161/15052249PJE2017.65.4.009](https://www.researchgate.net/profile/Agnieszka_Szlauer-Lukaszewska?_sg=AS_Hjn2c_XScF9N62HDuAb9yLvDcjXqp1wUkECjDVsVzDgr8ih_4MGcb0dn93tEvGeoTzQI.VVujEsx0xv_ZbBC9i53ID1d73exGub9HF9ecdIb4V40ptYb5gf_E4BqZxWreO8Ak3C8vK7RdhhGagP_kMw83IA) |
| Santos, M. J., Anderson, L. W., and Ustin S. L. (2011). Effects of invasive species on plant communities: An example using submersed aquatic plants at the regional scale. *Biol. Invasions.* 13, 443–457. doi: 10.1007/s10530-010-9840-6 |
| Schallenberg, M., and Sorrell, B. (2009). Regime shifts between clear and turbid water in New Zealand lakes: Environmental correlates and implications for management and restoration. *New. Zeal. J. Mar. Fresh.* 43, 701–712. doi: 10.1080/00288330909510035 |
| Silveira, M. J., Chollet, S., Thiébaut, G., and Thomaz, S. M. (2018). Abiotic factors, not herbivorous pressure, are primarily responsible for the performance of an invasive aquatic plant. *Ann. Limnol-Int. J. Lim*. 54, 12. doi: 10.1051/limn/2018002 |
| Sorte, C. J. B., Ibanez, I., Blumenthal, D. M., Molinari, N. A., Miller, L. P., Grosholz, E. D., Diez, J. M., D’Antonio, C. M., Olden, J. D., Jones, S. J., and Dukes, J. S. (2013). Poised to prosper? A cross-system comparison of climate change effects on native and non-native species performance. *Ecol. Lett.* 16, 261–270. doi: 10.1111/ele.12017 |
| Thouvenot, L., Haury, J., and Thiebaut, G. (2013). A success story: water primroses, aquatic plant pests. *Aquat. Conserv*. 23, 790–803. doi: 10.1002/aqc.2387 |
| Vojtko, A. E., Mesterhazy, A., Suveges, K., and Valk, O. (2017). Changes in sediment seed-bank composition of invaded macrophyte communities in a thermal river. *Freshwater. Biol*. 62, 1024–1035. doi: 10.1111/fwb.12922 |
| Wu, H., Ismail, M., and Ding, J. Q. (2017). Global warming increases the interspecific competitiveness of the invasive plant alligator weed, *Alternanthera philoxeroides*. *Sci. Total. Environ.* 575, 1415–1422. doi: 10.1016/j.scitotenv.2016.09.226 |
| You, W. H., Yu, D., Xie, D., Yu, L. F., Xiong, W., and Han, C. M. (2014). Responses of the invasive aquatic plant water hyacinth to altered nutrient levels under experimental warming in China. *Aqua. Bot.* 119, 51–56. doi: 10.1016/j.aquabot.2014.06.004 |

**Positive impacts of climatic warming (4 articles):**

| Dong, J., Yang, K., Li, S. S., Li, G. B., and Song, L. R. (2014). Submerged vegetation removal promotes shift of dominant phytoplankton functional groups in a eutrophic lake. *J. Environ. Sci-China.* 26, 1699–1707. doi: 10.1016/j.jes.2014.06.010 |
| --- |
| Hussner, A., and Losch, R. (2005). Alien aquatic plants in a thermally abnormal river and their assemblyto neophyte-dominated macrophyte stands (River Erft, Northrhine-Westphalia). *Limnologica.* 35, 18–30. doi: 10.1016/j.limno.2005.01.001 |
| Kolada, A., and Kutyia, S. (2016). *Elodea canadensis* (Michx.) in Polish lakes: a non-aggressive addition to native flora. *Biol. Invasions.* 18, 3251–3264. doi: 10.1007/s10530-016-1212-4 |
| Lu, X. M., Siemann, Evan., He, M. Y., Wei, H., Shao, X., and Ding, J. Q. (2016). Warming benefits a native species competing with an invasive congener in the presence of a biocontrol beetle. *New. Phytol.* 211, 1371–1381. doi: 10.1111/nph.13976 |

**Negative impacts of eutrophication (22 articles):**

| Bellinger, J. B. J., and Davis, S. L. (2017). Investigating the role of water and sediment chemistry from two reservoirs in regulating the growth potential of *Hydrilla verticillata* (L.f.) Royle and *Cabomba caroliniana* A. Gray. *Aquat. Bot.* 136, 175–185. doi: 10.1016/j.aquabot.2016.10.005 |
| --- |
| [Ceschin, S., Abati, S., Leacche, I., and Zuccarello, V. (2017). Ecological comparison between duckweeds in central Italy: The invasive Lemna minuta vs the native L. minor. Plant. Biosyst. 152, 674–683. doi: 10.1080/11263504.2017.1317671](https://doi.org/10.1080/11263504.2017.1317671) |
| Chase, J. M., and Knight, T. M. (2006). Effects of eutrophication and snails on Eurasian watermilfoil (*Myriophyllum spicatum*) invasion. *Biol. Invasions.* 8, 1643-1649. doi: 10.1007/s10530-005-3933-7 |
| Coetzee1, J. A., Hill, M. P., Byrne, M. J., Bownes, A. (2011). A review of the biological control programmes on *Eichhornia crassipes* (C.Mart.) Solms (Pontederiaceae), *Salvinia molesta* D.S.Mitch. (Salviniaceae), *Pistia stratiotes* L. (Araceae), *Myriophyllum aquaticum* (Vell.) Verdc. (Haloragaceae) and Azolla filiculoides Lam. (Azollaceae) in South Africa. *Afr. Entomol.* 19, 451–468. doi: 10.4001/003.019.0202 |
| Ding, W. J., Zhang, H. Y., Zhang, F. J., Wang, L. J., and Cui, S. B. (2014). Morphology of the invasive amphiphyte *Alternanthera philoxeroides* under different water levels and nitrogen concentrations. *Acta. Biol. Cracov. Bot.* 56, 136–147. doi: 10.2478/abcsb-2014-0028 |
| Espinar, J. L., Díaz-Delgado, R., Bravo-Utrera, M. A., and Vilà, M. (2015). Linking *Azolla filiculoides* invasion to increased winter temperature in the Doñana marshland (SW Spain). *Aquat. Invasions.* 10, 17–24. doi: 10.3391/ai.2015.10.1.02 |
| Gerard, J., and Triest, L. (2018). Competition between invasive *Lemna minuta* and native *L. minor* in indoor and field experiments. *Hydrobiologia.* 812, 57–65. doi: 10.1007/s10750-016-2754-2 |
| Gérard, J., Brion, N., and Triest L. (2014). Effect of water column phosphorus reduction on competitive outcome and traits of *Ludwigia grandiflora* and *L. peploides*, invasive species in Europe. *Aquat. Invasions.* 9, 157–166. doi: 10.3391/ai.2014.9.2.04 |
| Graeme, T., Hastwell, A. J., and Daniel, G. V. S. (2008). Predicting invasiveness in exotic species: do subtropical native and invasive exotic aquatic plants differ in their growth responses to macronutrients? *Divers. Distrib.* 14, 243–251. doi: 10.1111/j.1472-4642.2007.00367.x |
| Hussner, A., and Losch, R. (2007). Growth and photosynthesis of *Hydrocotyle ranunculoides* L. fil. in Central Europe. *Flora.* 202, 653–660. doi: 10.1016/j.flora.2007.05.006 |
| Li, W. G., and Wang, J. B. (2011). Influence of light and nitrate assimilation on the growth strategy in clonal weed *Eichhornia crassipes*. *Aquat. Ecol.* 45, 1–9. doi: 10.1007/s10452-010-9318-8 |
| Loo, S. E., Mac Nally, R., O’Dowd, D. J., Thomson, J. R., and Lake, P. S. (2009). Multiple scale analysis of factors influencing the distribution of an invasive aquatic grass. *Biol. Invasions.* 11, 1903–1912. doi: 10.1007/s10530-008-9368-1 |
| McElarney, Y. R., Rasmussen, P., Foy, R.H., and Anderson, N. J. (2010). Response of aquatic macrophytes in Northern Irish softwater lakes to forestry management; eutrophication and dissolved organic carbon. *Aquat. Bot.* 93, 227–236. doi: 10.1016/j.aquabot.2010.09.002 |
| Quinn, L. D., Schooler, S. S., and van Klinken, R. D. (2010). Effects of land use and environment on alien and native macrophytes: lessons from a large-scale survey of Australian rivers. *Divers. Distrib.* 17, 132–143. doi: 10.1111/j.1472-4642.2010.00726.x |
| [Ranta, P., and Toivonen, H. (2008). Changes in aquatic macrophytes since 1933 in an urban lake, lidesjärvi, SW Finland. Ann. Bot. Fenn. 45, 359–371. doi: 10.5735/085.045.0503](https://hub3.qboshi.net/scholar_url?url=https%3A%2F%2Fwww.jstor.org%2Fstable%2F23727700&hl=zh-CN&sa=T&ct=res&cd=0&d=10464334635744272612&ei=9QJ9W_vjOtWLyQTAmbnwDg&scisig=AAGBfm3mD7v7C3z1DBp149VnI_D85GDmoQ&nossl=1&ws=1067x527) |
| Riis, T., Lambertini, C., Olesen, B., Clayton, J. S., Brix, H., and Sorrell, B. K. (2010). Invasion strategies in clonal aquatic plants: are phenotypic differences caused by phenotypic plasticity or local adaptation? Ann. Bot-London. 106, 813–822. doi: 10.1093/aob/mcq176 |
| Thiebaut, G. (2005). Does competition for phosphate supply explain the invasion pattern of Elodea species? *Water. Res.* 39, 3385–3393. doi: 10.1016/j.watres.2005.05.036 |
| Thomsen, M. S., McGlathery, K. J. (2007). Stress tolerance of the invasive macroalgae *Codium fragile* and *Gracilaria vermiculophylla* in a soft-bottom turbid lagoon. *Biol. Invasions.* 9, 499–513. doi: 10.1007/s10530-006-9043-3 |
| Thouvenot, L., Haury, J., and Thiebaut, G. (2013). A success story: water primroses, aquatic plant pests. *Aquat. Conserv*. 23, 790–803. doi: 10.1002/aqc.2387 |
| Wersal, R. M., and Madsen, J. D. (2011). Influences of water column nutrient loading on growth characteristics of the invasive aquatic macrophyte *Myriophyllum aquaticum* (Vell.) Verdc. *Hydrobiologia*. 665, 93–105. doi: 10.1007/s10750-011-0607-6 |
| You, W. H., Yu, D., Xie, D., Yu, L. F., Xiong, W., and Han, C. M. (2014). Responses of the invasive aquatic plant water hyacinth to altered nutrient levels under experimental warming in China. *Aquat. Bot.* 119, 51–56. doi: 10.1016/j.aquabot.2014.06.004 |
| [Yu, H. H., Wang, L. G., Liu, C. H., and Fan, S. F. (2018). Coverage of native plants is key factor influencing the invasibility of freshwater ecosystems by exotic plants in China. Front. Plant. Sci. 9, 250. doi: 10.3389/fpls.2018.00250](http://xueshu.baidu.com/usercenter/data/journal?cmd=jump&wd=journaluri%3A%28b8005f4a9d2fc822%29%20%E3%80%8AFrontiers%20in%20Plant%20Science%E3%80%8B&tn=SE_baiduxueshu_c1gjeupa&ie=utf-8&sc_f_para=sc_hilight%3Dpublish&sort=sc_cited) |

**Positive impacts of eutrophication (6 articles):**

| Qin, H. J., Zhang, Z. Y., Liu, M. H., Liu, H. Q., Wang, Y., Wen, X. Z., Zhang, Y. Y., Yan, S. H. (2016). Site test of phytoremediation of an open pond contaminated withdomestic sewage using water hyacinth and water lettuce. *Ecol. Eng.* 95, 753–762. doi: 10.1016/j.ecoleng.2016.07.022 |
| --- |
| Brouwer, E., Denys, L., Lucassen, E. C., Buiks, M., and Onkelinx, T. (2017) Competitive strength of Australian swamp stonecrop (*Crassula helmsii*) invading moorland pools. *Aquat. Invasions.* 12, 321–331. doi: 10.3391/ai.2017.12.3.06 |
| Kolada, A., and Kutyła, S. (2016). *Elodea canadensis* (Michx.) in Polish lakes: a non-aggressive addition to native flora. *Biol. Invasions.* 18, 3251–3264. doi: 10.1007/s10530-016-1212-4 |
| Wu, H., Carrillo, J., Ding, J. Q. (2017). Species diversity and environmental determinants of aquatic and terrestrial communities invaded by *Alternanthera philoxeroides*. *Sci. Total. Environ.* 581–582, 666–675. doi: 10.1016/j.scitotenv.2016.12.177 |
| [Xu, X., Yang, L., Huang, X. L., Li, Z. Q., Yu, D. (2017). Water brownification may not promote invasions of submerged non-native macrophytes. Hydrobiologia. 817, 215–225. doi: 10.1007/s10750-017-3387-9](http://xueshu.baidu.com/usercenter/data/journal?cmd=jump&wd=journaluri%3A%286066b5550bb4d6cf%29%20%E3%80%8AHydrobiologia%E3%80%8B&tn=SE_baiduxueshu_c1gjeupa&ie=utf-8&sc_f_para=sc_hilight%3Dpublish&sort=sc_cited) |
| Zuo, S. P., Ma, Y. Q., and Shinobu, I. (2012). Differences in ecological and allelopathic traits among *Alternanthera philoxeroides* populations. *Weed. Biol. Manag.* 12, 123–130. doi: 10.1111/j.1445-6664.2012.00443.x |

**Negative impacts of elevated rainfall (18 articles):**

| Adamec, L., and Lev, J. (1999). The introduction of the aquatic carnivorous plant *Aldrovanda vesiculosa* to new potential sites in the Czech Republic: A five-year investigation. *Folia. Geobot.* 34, 299–305. doi: 10.2307/4201379 |
| --- |
| Anufriieva, E. V., and Shadrin, N. V. (2017). Extreme hydrological events destabilize aquatic ecosystems and open doors for alien species. *Quatern. Int.*475, 11–15. DOI: 10.1016/j.quaint.2017.12.006 |
| Coetzee1, J. A., Hill, M. P., Byrne, M. J., Bownes, A. (2011). A review of the biological control programmes on *Eichhornia crassipes* (C.Mart.) Solms (Pontederiaceae), *Salvinia molesta* D.S.Mitch. (Salviniaceae), *Pistia stratiotes* L. (Araceae), *Myriophyllum aquaticum* (Vell.) Verdc. (Haloragaceae) and Azolla filiculoides Lam. (Azollaceae) in South Africa. *Afr. Entomol.* 19, 451–468. doi: 10.4001/003.019.0202 |
| Collinge, S. K., Ray, C., and Gerhardt, F. (2011). Long-term dynamics of biotic and abiotic resistance to exotic species invasion in restored vernal pool plant communities. *Ecol. Appl.* 21, 2105–2118. doi: 10.2307/41416641 |
| Detenbeck, N. E., Galatowitsch, S. M., Atkinson, J., Ball, H. (1999). Evaluating perturbations and developing restoration strategies for inland wetlands in the Great Lakes basin. *Wetlands.* 19, 789–820. doi: 10.1007/BF03161785 |
| Espinar, J. L., Díaz-Delgado, R., Bravo-Utrera, M. A., and Vilà, M. (2015). Linking *Azolla filiculoides* invasion to increased winter temperature in the Doñana marshland (SW Spain). *Aquat. Invasions.* 10, 17–24. doi: 10.3391/ai.2015.10.1.02 |
| Hofstra, D., Champion, P., and Clayton, J. (2010). Predicting invasive success of *Hydrilla verticillata* (L.f.) Royle in flowing water. *Hydrobiologia.* 656, 213–219. doi: 10.1007/s10750-010-0445-y |
| Holmes, P. M., Esler, K. J., Richardson, D. M., Witkowski, E. T. F. (2008). Guidelines for improved management of riparian zones invaded by alien plants in South Africa. *S. Afr. J. Bot.* 74, 538–552. doi: 10.1016/j.sajb.2008.01.182 |
| Keller, R. P., Masoodi, A., and Shackleton, R. T. (2018). The impact of invasive aquatic plants on ecosystem services and human well-being in Wular Lake, India. *Reg. Environ. Change.*18, 847–857. doi: 10.1007/s10113-017-1232-3 |
| Mugwedi, L. F., Goodall, J., Witkowski, E. T. F., and Byrne, M. J. (2015). The role of reproduction in *Glyceria maxima* invasion. Afr. J. Range. For. Sci. 32, 59–66. doi: 10.2989/10220119.2014.929177 |
| O’Meara, G. F., Cutwa, M. M., Jr Evans, L. F. (2003). Bromeliad-inhabiting mosquitoes in south Florida: native and exotic plants differ in species composition. *J. Vector. Ecol.* 28, 37–46. |
| Rodríguez-Merino, A., Fernández-Zamudio, R., and García-Murillo, P. (2017). An invasion risk map for non-native aquatic macrophytes of the Iberian Peninsula. *An. Jardin. Bot. Madrid.* 74, e055. doi: 10.3989/ajbm.2452 |
| Sousa, W. T. Z., Thomaz, S. M., and Murphy, K. J. (2010). Response of native *Egeria najas* Planch. and invasive *Hydrilla verticillata* (L.f.) Royle to altered hydroecological regime in a subtropical river. *Aquat. Bot.* 92, 40–48. doi: 10.1016/j.aquabot.2009.10.002 |
| Swanson, W., De Jager, N. R., Strauss, E., and Thomsen, M. (2017). Effects of flood inundation and invasion by *Phalaris arundinacea* on nitrogen cycling in an Upper Mississippi River floodplain forest. *Ecohydrology.*10, e1877. doi: 10.1002/eco.1877 |
| Thomas, J. R., Gibson, D. J., and Middleton, B. A. (2005). Water dispersal of vegetative bulbils of the invasive exotic *Dioscorea oppositifolia* L. in southern Illinois. *J. Torrey. Bot. Soc.* 132, 187–196. doi: 10.3159/1095-5674 |
| Thouvenot, L., Haury, J., and Thiebaut, G. (2013). A success story: water primroses, aquatic plant pests. *Aquat. Conserv*. 23, 790–803. doi: 10.1002/aqc.2387 |
| Wu, H., Carrillo, J., Ding, J. Q. (2017). Species diversity and environmental determinants of aquatic and terrestrial communities invaded by *Alternanthera philoxeroides*. Sci. Total. Environ. 581–582, 666–675. doi: 10.1016/j.scitotenv.2016.12.177 |
| You, W. H., Yu, D., Liu, C. H., Xie, D., and Xiong, W. (2013). Clonal integration facilitates invasiveness of the alien aquatic plant *Myriophyllum aquaticum* L. under heterogeneous water availability. *Hydrobiologia*. 718, 27–39. DOI: 10.1007/s10750-013-1596-4 |

**Positive impact of elevated rainfall (1 article):**

Lamb, H. F., Darbyshire, I., and Verschuren, D. (2003). Vegetation response to rainfall variation and human impact in central Kenya during the past 1100 years. *Holocene.* 13, 285–292. doi: 10.1191/0959683603hl618rr

**Negative impact of global trade (31 articles):**

| Adebayo, A. A., Briski, E., Kalaci, O., Hernandez, M., Ghabooli, S., Beric, B., Chan, F. T., Zhan, A. B., Fifield, E., Leadley, E. T., and MacIsaac, H. J. (2011) Water hyacinth (*Eichhornia crassipes*) and water lettuce (*Pistia stratiotes*) in the Great Lakes: playing with fire? *Aquat. Invasions.* 6, 91–96. doi: 10.3391/ai.2011.6.1.11 |
| --- |
| Andreu, J., and Vila, M. (2010). Risk analysis of potential invasive plants in Spain. *J. Nat. Conserv.* 18, 34–44. doi: 10.1016/j.jnc.2009.02.002 |
| Ardenghi, N. M. G., Armstrong, W. P., and Paganelli, D. (2017). *Wolffia columbiana* (Araceae, Lemnoideae): first record of the smallest alien flowering plant in southern Europe and Italy. *Bot. Lett.*164, 121–127. doi: 10.1080/23818107.2017.1319293 |
| [Ardenghi, N. M. G., Barcheri, G., Ballerini, C., Cauzzi, P., and Guzzon, F. (2016). Gymnocoronis spilanthoides (Asteraceae, Eupatorieae), a new naturalized and potentially invasive aquatic alien in S Europe. Willdenowia. 46, 265–273. DOI: 10.3372/wi.46.46208](http://www.bioone.org/doi/abs/10.3372/wi.46.46208) |
| Azan, S., Bardecki, M., and Laursen, A. E. (2015). Invasive aquatic plants in the aquarium and ornamental pond industries: a risk assessment for southern Ontario (Canada). *Weed. Res.* 55, 249–259. |
| Brundu, G. (2015). Plant invaders in European and Mediterranean inland waters: profiles, distribution, and threats. *Hydrobiologia*. 746, 61–79. doi: 10.1007/s10750-014-1910-9 |
| Brundu, G., Azzella, M. M., Blasi, C., Camarda, I., Iberite, M., and Celesti-Grapow L. (2013). The silent invasion of *Eichhornia crassipes* (Mart.) Solms. in Italy. *Plant. Biosystems*. 147, 1120–1127. Doi: 10.1080/11263504.2013.861536 |
| Champion, P. D., Clayton, J. S., and Hofstra, D. E. (2010). Nipping aquatic plant invasions in the bud: weed risk assessment and the trade. *Hydrobiologia*. 656, 167–172. doi: 10.1007/s10750-010-0446-x |
| Ding, J. Q., Mack, R. N., Lu, P., Ren, M. X., and Huang, H. W. (2008). China’s booming economy is sparking and accelerating biological invasions. *Bioscience.* 58, 317–324. doi: 10.1641/B580407 |
| Essl, F., Dullinger, S., Rabitsch, W., Hulme, P. E., Hülber, K., Jarošík, V., Kleinbauer, I., Krausmann, F., Kühn, I., Nentwig, W., Vilà, M., Genovesi, P., Gherardi, F., Desprez-Loustau, M. L., Roques, A., and Pyšek, P. (2011). Socioeconomic legacy yields an invasion debt. *PNAS.* 108, 203–207. doi: 10.1073/pnas.1011728108 |
| Ghahramanzadeh, R., Esselink, G., Kodde, L. P., Duistermaat, H., Van Valkenburg, J. L. C. H., Marashi, S. H., Smulders, M. J. M., and Van de Wiel, C. C. M. (2012). Efficient distinction of invasive aquatic plant species from non-invasive related species using DNA barcoding. *Mol. Ecol. Resour.* 13, 21–31. doi: 10.1016/j.aquabot.2015.09.002 |
| Hoveka, L. N., van der Bank, M., Boatwright, J. S., Bezeng, B. S., Yessoufou, K. (2016). The noncoding trnH-psbA spacer, as an effective DNA barcode for aquatic freshwater plants, reveals prohibited invasive species in aquarium trade in South Africa. *S. Afr. J. Bot.* 102, 208–216. Doi: 10.1016/j.sajb.2015.06.014 |
| Hussner, A., Nehring, S., Hilt, S. (2014). From first reports to successful control: a plea for improved management of alien aquatic plant species in Germany. Hydrobiologia. 737, 321–331. doi: 10.1007/s10750-013-1757-5 |
| June-Wells, M., Vossbrinck, C. R., Gibbons, J., et al. (2012). The aquarium trade: A potential risk for nonnative plant introductions in Connecticut, USA. *Lake. Reserv. Manage.* 28, 200–205. doi: 10.1080/07438141.2012.693575 |
| Maki, K., and Galatowitsch, S. (2004). Movement of invasive aquatic plants into Minnesota (USA) through horticultural trade. *Biol. Conserv.* 118, 389–396. doi: 10.1016/j.biocon.2003.09.015 |
| [Martin, G. D., and Coetzee, J. A. (2011). Pet stores, aquarists and the internet trade as modes of introduction and spread of invasive macrophytes in South Africa. Water. S. A. 37, 371–380. doi: 10.4314/wsa.v37i3.68488](http://xueshu.baidu.com/usercenter/data/journal?cmd=jump&wd=journaluri%3A%289e6fc38131dbaa94%29%20%E3%80%8AWater%20S%20A%E3%80%8B&tn=SE_baiduxueshu_c1gjeupa&ie=utf-8&sc_f_para=sc_hilight%3Dpublish&sort=sc_cited) |
| Minchin, D, and Boelens, R. (2011). The distribution and expansion of ornamental plants on the Shannon navigation. *Biol. Environ: P. Ro. Irish Acad.* 111B, 195–203. doi: 10.2307/23188048 |
| Ng, T. H., Limpanont, Y., Chusongsang,Y., Chusongsang, P., Panha, S (2018). Correcting misidentifications and first confirmation of the globally-invasive Physa acuta Draparnaud, 1805 (*Gastropoda: Physidae*) in Thailand and Laos. *BioInvasions. Rec.* 7, 15–19. doi: 10.3391/bir.2018.7.1.03 |
| Nierbauer, K. U., Kanz, B., Zizka, G. (2014). The widespread naturalisation of Nymphaea hybrids is masking the decline of wild-type *Nymphaea alba* in Hesse, Germany. *Flora.* 209, 122–130. doi: 10.1016/j.flora.2013.12.005 |
| Oertli, B., Boissezon, A., Rosset, V., Ilg, C. (2017). Alien aquatic plants in wetlands of a large European city (Geneva, Switzerland): from diagnosis to risk assessment. *Urban. Ecosyst.* doi: 10.1007/s11252-017-0719-5. |
| Patoka, J., Bláha, M., Kalous L, Kouba, A. (2017). Irresponsible vendors: Non-native, invasive and threatened animals offered for garden pond stocking. *Aquatic. Conserv.* 27, 692–697. doi: 10.1002/aqc.2719 |
| Pyšek, P., Jarošík, V., Hulme, P. E., Kühn, I., Wild, J., Arianoutsou, M., Bacher, S., Chiron, F., Didžiulis, V., Essl F.,…Winter M. (2010). Disentangling the role of environmental and human pressures on biological invasions across Europe. *PNAS.* 27, 12157–12162. doi: 10.1073/pnas.1002314107 |
| Patoka, J., Bláha, M., Kalous, L., Vrabec, V., Buřič, M., and Kouba, A. (2016). Potential pest transfer mediated by international ornamental plant trade. *Sci. Rep.* 6, 25896. doi: 10.1038/srep25896 |
| Robert, W. (2009). Pemberton, Hong Liu. Marketing time predicts naturalization of horticultural plants. *Ecology.* 90, 69–80. doi: 10.1890/07-1516.1 |
| Snyder, E., Francis, A., and Darbyshire, S. J. (2016). Biology of invasive alien plants in Canada. 13. *Stratiotes aloides* L. *Can. J. Plant Sci.* 96, 225–242. doi: 10.1139/cjps-2015-0188 |
| Tamayo, M., and Olden, J. D. (2014). Forecasting the vulnerability of lakes to aquatic plant invasions. *Invas. Plant. Sci. Mana.* 7, 32–45. doi: 10.1614/IPSM-D-13-00036.1 |
| Thum, R. A., Mercer, A. T., and Wcisel, D. J. (2012). Loopholes in the regulation of invasive species: genetic identifications identify mislabeling of prohibited aquarium Plants. Biol Invasions, 2012, 14: 929–937. doi: 10.1007/s10530-011-0130-8 |
| Vasconcelos, T., Tavares, M., and Gaspar, N. (1999). Aquatic plants in the rice fields of the Tagus Valley, Portugal. *Hydrobiologia*. 415, 59–65. doi: 10.1023/A:1003873315570 |
| Wasekura, H., Horie, S., Fujii, S., and Maki, M. (2016). Molecular identification of alien species of Vallisneria (Hydrocharitaceae) species in Japan with a special emphasis on thecommercially traded accessions and the discovery of hybrid between non indigenous *V. spiralis* and native *V. denseserrulata*. *Aquat. Bot.* 128, 1–6. doi: 10.1016/j.aquabot.2015.09.002 |
| Yanai, Z., Dayan, T., Mienis, H. K., Gasith, A. (2017). The pet and horticultural trades as introduction and dispersal agents of non-indigenous freshwater molluscs. *Manag. Biol. Invasion.* 8, 523–532. |
